# Continuous Monitoring of Urine Output And Hemodynamic Disturbances Improves Early Detection of Acute Kidney Injury During First Week of Icu Stay

**DOI:** 10.1186/2197-425X-3-S1-A13

**Published:** 2015-10-01

**Authors:** M Flechet, F Güiza, M Schetz, P Wouters, I Vanhorebeek, J Gunst, G Van den Berghe, G Meyfroidt

**Affiliations:** KULeuven, Laboratory of Intensive Care Medicine, Leuven, Belgium

## Introduction

Acute Kidney Injury (AKI) is associated with increased morbidity and mortality in critically ill patients [1]. Early detection and treatment may improve outcome. Previously, we developed a logistic regression (LR) model for early detection of AKI based on routinely collected data available at baseline, ICU admission and at the end of the first day (LR_BAD1) [2]. Continuous monitoring parameters may provide additional predictive power, in particular, urine output and hemodynamic parameters, whose management influences kidney perfusion.

## Objectives

To assess if adding continuous monitoring variables recorded during the first 24h of ICU stay, to a model to predict AKI in the first week of ICU admission, can improve the predictive performance.

## Methods

The model was built and validated in a subset of 1778 ICU patients from the EPaNIC trial [3]. Patients with end-stage renal disease, those with AKI on the first day of ICU stay and those without available hemodynamic monitoring data during the first day were excluded. AKI was defined by the creatinine criteria from the KDIGO guidelines.

The LR_BAD1 model used only covariates selected at baseline, ICU admission and at the end of the first day. in the LR_BAD1+ model, we have added features extracted from hourly measures of urine and minute-by-minute measures of heart frequency (HF) and mean arterial blood pressure (MABP). Moreover, the cumulative dose of inotropes administered to each patient during the first 24h of ICU stay was used as additional covariate. The predictive power was assessed by ROC, decision and calibration curves analysis using 300 bootstraps replicates.

## Results

**Figure 1:**
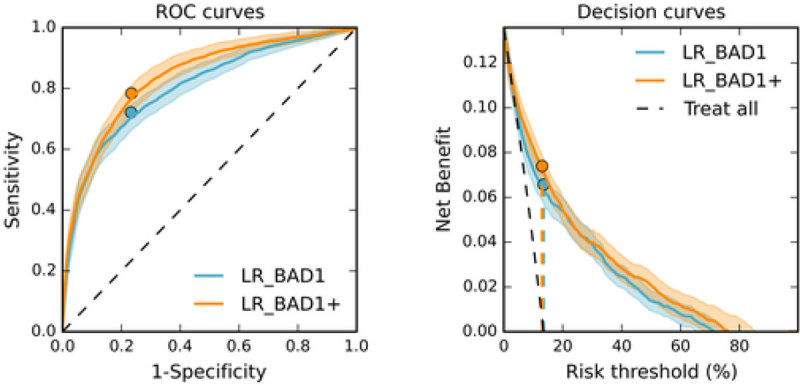
**ROC and decision curves of LR_BAD1 and LR_BAD1+.**

Baseline characteristics are reported in Table 1. Performance of the model is shown on Figure 1 and in Table 2. Performance of LR-BAD1 is slightly different than what was reported in [2] as here it is evaluated in a different population. As compared to the LR-BAD1 model, the LR-BAD1+ model had better discrimination (Auroc p-value = 0.05 and discrimination slope p-value < 0.01) while retaining good calibration and net benefit. Model performance increased the most with hemodynamics features. Highly predictive features included the MABP median and slope, the time the patient's MABP was outside the interval defined by the MABP mean +- 2 standard deviation, HF median and standard deviation, the total amount of urine and the number of urine measurements.

**Table 1:** Demographics - 1778 ICU patients.

Age (years): median [IQR]	65.50 [54.55-74.50]
Apache II: median [IQR]	18.00 [13.00-29.00]
ICU length of stay (hours): median [IQR]	69.00 [35.00-151.00]
Baseline serum creatinine (mg/dl): median [IQR]	0.92 [0.77-1.06]
Type of admission: elective/emergency (%)	1155 (64.96) / 623 (35.04)
Diagnostic group: cardiac/transplant/non cardiac surgery and trauma-burns/medical-others (%)	1186 (66.70) / 131 (7.37) / 385 (21.65) / 76 (4.27)
Male gender: n (%)	1123 (63.16)
Sepsis on ICU admission: n (%)	285 (16.03)
Diabetes: n (%)	274 (15.41)
Incidence of AKI within first week: n (%)	241 (13.55)

**Table 2:** Statistics of LR_BAD1 and LR_BAD1+.

	LR_BAD1	LR_BAD1+	p-value
Auroc	0.80 +- 0.01	0.84 +- 0.01	0.05
Net benefit	0.07 +- 0.01	0.08 +- 0.01	0.20
Calibration slope	0.87 +- 0.09	0.88 +- 0.08	0.45
Calibration-in-the-large	0.00 +- 0.01	0.00 +- 0.01	0.47
Discrimination slope	0.22 +- 0.01	0.27 +- 0.02	< 0.01

## Conclusions

Early detection of AKI can be improved by routinely monitored information of urine and hemodynamics. Hence, AKI can be already predicted with high discrimination and good calibration, only by using routinely collected ICU data.

## Grant Acknowledgment

GM receives funding from FWO (1846113N). GVdB receives long-term research financing via the Flemish government Methusalem-program.
